# An Improved LDA-Based ELM Classification for Intrusion Detection Algorithm in IoT Application

**DOI:** 10.3390/s20061706

**Published:** 2020-03-19

**Authors:** Dehua Zheng, Zhen Hong, Ning Wang, Ping Chen

**Affiliations:** 1 Faculty of Mechanical Engineering & Automation, Zhejiang Sci-Tech University, Hangzhou 310018, China; zhengdehua6666@gmail.com; 2 College of Information Engineering, Zhejiang University of Technology, Hangzhou 310023, China; 3 School of Electrical & Computer Engineering, Georgia Institute of Technology, Atlanta, GA 30332, USA; 4 School of Public Administration, Zhejiang University of Finance & Economics, Hangzhou 310018, China; 5 Zhejiang Provincial Testing Institute of Electronic Information Products, Hangzhou 310007, China

**Keywords:** IoT, intrusion detection, linear discriminant analysis, extreme learning machine, classification

## Abstract

The Internet of Things (IoT) is widely applied in modern human life, e.g., smart home and intelligent transportation. However, it is vulnerable to malicious attacks, and the current existing security mechanisms cannot completely protect the IoT. As a security technology, intrusion detection can defend IoT devices from most malicious attacks. However, unfortunately the traditional intrusion detection models have defects in terms of time efficiency and detection efficiency. Therefore, in this paper, we propose an improved linear discriminant analysis (LDA)-based extreme learning machine (ELM) classification for the intrusion detection algorithm (ILECA). First, we improve the linear discriminant analysis (LDA) and then use it to reduce the feature dimensions. Moreover, we use a single hidden layer neural network extreme learning machine (ELM) algorithm to classify the dimensionality-reduced data. Considering the high requirement of IoT devices for detection efficiency, our scheme not only ensures the accuracy of intrusion detection, but also improves the execution efficiency, which can quickly identify the intrusion. Finally, we conduct experiments on the NSL-KDD dataset. The evaluation results show that the proposed ILECA has good generalization and real-time characteristics, and the detection accuracy is up to 92.35%, which is better than other typical algorithms.

## 1. Introduction

**Figure 1:**
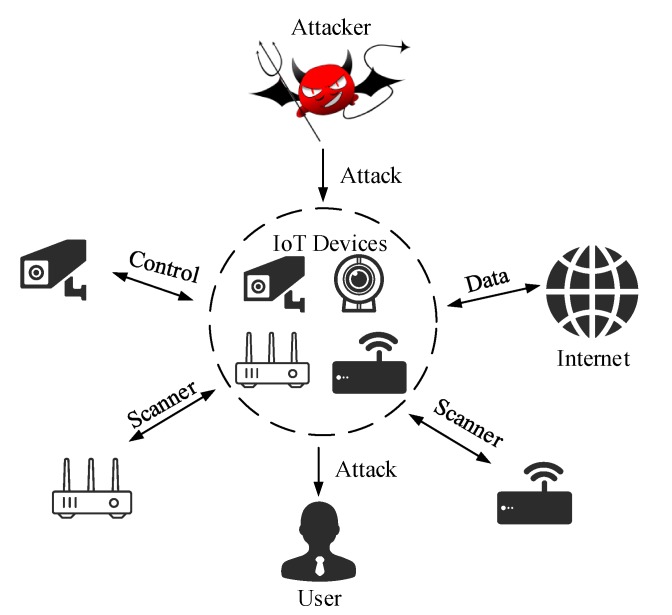
Internet of Things (IoT) attack.

The Internet of Things (IoT) is an extension of the traditional network that combines various sensing devices with the Internet. Recently, IoT is also increasingly used in production and human life in applications such as smart plant, smart home, intelligent transportation, battlefield monitoring, medical facility, and environmental monitoring [1,2,3]. The security of IoT has gradually attracted widespread attention. As shown in Figure 1, once IoT is maliciously attacked, it is likely to suffer significant losses. IoT devices have the disadvantage of limited resources characterized by weak computing power, low memory space, and unattended. This makes the information confidentiality and integrity of IoT devices during transmission of data seriously threatened [4]. Therefore, the security of the data generated from various IoT devices is one of the biggest concerns [5]. The defenders establish the first line of defense for IoT devices through security techniques such as cryptographic authentication and secure network topology construction. However, some attackers still launch malicious attacks on IoT devices through data analysis. Intrusion detection is considered as the second line of defense, which plays an important role in ensuring the security of IoT devices [6]. Intrusion detection detects and identifies potential attack behaviors in IoT devices, which makes correct responses in time and implements effective protection measures. For example, the Markov model, Intrusion Detection System (IDS), and Virtual Honeypot Device (VHD) are used to identify malicious edge devices in a fog computing environment, thereby protecting the system from unauthenticated devices [7]. Therefore, the building of a reasonable intrusion detection system (IDS) for IoT applications is a research hotspot in the field of IoT security, and, specifically, its algorithms are the core of the current research on IDS.

Usually, the intrusion detection system (IDS) is used to analyze and judge the collected network data. IDS detects whether there is an attack behavior in the network and immediately performs response processing. Recently, a large number of techniques are applied to intrusion detection, such as rule-based network intrusion detection technology [8], artificial immune system [9], clustering-based technology [10,11], Support Vector Machine (SVM) [12,13], neural network [14,15,16], etc. Currently, among many intrusion detection algorithms, neural network-related algorithms have been widely used because of their good robustness and adaptability.

Manzoor et al. [17] proposed a feature-based dimensionality-reduced Artificial Neural Network (ANN) classifier for high-dimensional problems in IDS. First, the method proposes ranking of the obtained feature information gain and correlation. Then, it combines them to calculate the feature’s information degree, and chooses to retain the features that have greater effect on data classification. Finally, the system uses an ANN classifier to classify the data. This method not only eliminates redundant and irrelevant data using preprocessing, but also improves resource utilization and reduces time complexity.

However, for intrusion detection with a large amount of data, the time complexity of traditional neural network algorithms is too high to be suitable for IoT. Aburomman et al. [18] proposed a weighted one-against-rest SVM (WOAR-SVM) classifier algorithm after comparing multiple SVM-based classifier models. This method uses a set of weights to compensate a single binary classifier, and each binary classifier has its own unique set of classification parameters. Finally, two classifiers are used to classify and predict a multi-class classifier. The experimental results show that WOAR-SVM has outstanding performance in the overall prediction accuracy of the multi-class data.

Zhang et al. [19] proposed a new method for intrusion detection using a probabilistic neural network (PNN). This method only requires a feedforward process and does not require backpropagation. Compared with naive Bayes and the back propagation (BP) neural network, the training time is greatly shortened. Brown et al. [20] aimed at achieving balance between the detection rate and false detection rate in IDS, and improved the general regression neural network through parameter optimization as an intrusion detection algorithm. Experiments show that this parameter optimization reduces the computational complexity and improves accuracy.

Although the above methods have better detection accuracy in intrusion detection, they also have common defects, such as high computational complexity and high time cost for intrusion detection. To address the problem of time efficiency, an efficient single-layer feedforward neural network extreme learning machine is initially introduced into IDS [21]. The extreme learning machine (ELM) [22] is an algorithm proposed in 2006 to solve a single hidden layer neural network. Its high prediction accuracy and efficient learning speed in classification algorithms have attracted widespread attention.

Cheng et al. [21] proposed an IDS based on a weighted ELM, which is mainly used for the classification of imbalanced classes. In light of the fact that intrusion data often occupies a small part or different attack data distribution, the system uses weighted ELM to perform intrusion detection, thereby improving the accuracy of intrusion detection. Singh et al. [23] proposed the IDS by combining three stages: feature selection, trust calculation, and classification decision. This method comprehensively examines the security of the nodes, so as to improve the accuracy of the network’s intrusion detection. However, its comprehensiveness makes the cost of resources high. Singh et al. [24] propose an intrusion detection technology based on online sequential ELM in response to the challenges of large data volume and high false positive rate in intrusion detection.

As a result of the expansion of the scale of IoT devices, the collected data presents multiple features, complex structures, and high feature dimensions. This makes the existing intrusion detection algorithms degradation of real-time and detection performance for such high-dimensional feature data.

To address the above problems, we propose an improved LDA-based ELM classification for the intrusion detection algorithm (namely, ILECA) that achieves rapid and efficient detection of attacks. In ILECA, we improve the linear discriminant analysis (LDA) and add a spatial similarity function to make high-dimensional data obtain a better spatial separation after dimensionality reduction. Subsequently, we use the extreme learning machine (ELM) classifier with fast classification to make the final decision for intrusion detection on the dimensionality-reduced data. The major contributions of this paper are summarized as follows.

(1) For IoT intrusion detection, we use the similarity measure function of high-dimensional data space as the weight to improve the between-class scatter matrix, and then combine it with LDA to obtain the optimal transformation matrix to achieve the dimensionality reduction of the data. The addition of the similarity measure function of high-dimensional data space gives the data better spatial separation, thereby improving the dimensionality reduction performance.
(2) We use the ELM classification to classify and detect the data after dimension reduction. ELM classification indeed helps speed up the overall learning rate of the algorithm as well as strengthening the capabilities of generalization. After evaluation tests using NSL-KDD, the accuracy and detection rate of our algorithm are, respectively, up to 92.35% and 91.53%, but its runtime is only 0.1632 s, of which the overall performance is better than the other five typical algorithms.

The rest of this paper is organized as follows. We simply introduce the intrusion detection in Section 2. We also propose an improved LDA-based ELM classification for intrusion detection algorithm with its details in Section 3. Furthermore, the performance evaluation is conducted to see the effectiveness of the proposed scheme in Section 4. We conclude this paper and show our future work in Section 5.

## 2. Intrusion Detection Model

**Figure 2:**
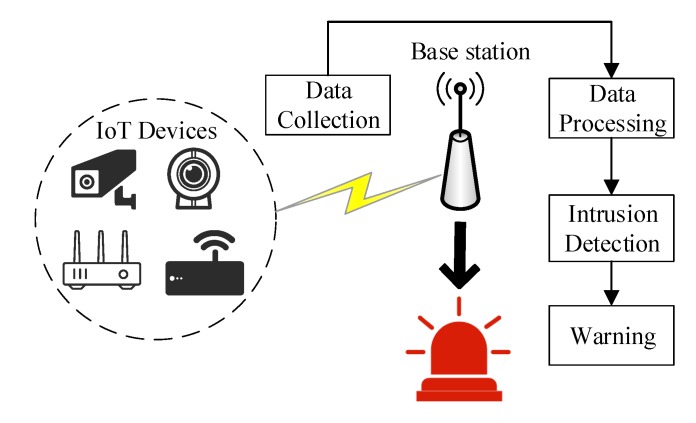
Intrusion detection model.

To protect the security of IoT devices, it is necessary to build an IDS for IoT devices. However, due to the limited resources of IoT devices, if an IDS is installed on all IoT devices, it will undoubtedly put greater pressure on the IoT devices. Moreover, it also leads to a sharp decline in the device lifetime, which increases device costs. Therefore, we mainly complete intrusion detection functions at the base station, which has a high ability in computing and storage. The intrusion detection model is shown in Figure 2.

The model mainly includes a data acquisition module, a data processing module, an intrusion detection algorithm, and an alarm module. The intrusion detection model has the requirements of high detection accuracy and high real-time performance. To ensure the security of the IoT and realize the rapid and efficient network intrusion detection, we mainly focus on the detection algorithms of the intrusion detection module. After collecting the data, we add the spatial similarity function of the high-dimensional data to the linear discriminant analysis method to reduce the dimension. This results in better spatial separation between classes after dimensionality reduction. Then, we use the extreme learning machine classifier to realize the final intrusion detection to determine whether the network is under attack as well as the corresponding attack type.

## 3. The Proposed ILECA

### 3.1. The Correlated Variables

Table 1 shows the correlated variables of ILECA. Specifically, they are divided into three categories: the dataset, the correlation variables of data dimensionality reduction, and the correlation variables of the neural network.

### 3.2. Data Preprocessing

Generally, we can perform intrusion detection through data analysis. The data is classified into two categories: continuous data and discrete data. Here, different data categories should be performed with different operations.

For continuous data, to reduce the impact of the different data dimensions on experimental results, the data is usually normalized before data reduction and classification. Normalization is the process of transforming a dimensionless expression into a dimensionless expression. When using principal component analysis (PCA) or the LDA algorithm for data dimensionality reduction, a covariance calculation is often involved. Among them, the Z-score normalization method can eliminate the effects of dimensional variance and covariance. It performs better than other normalization methods, so we use the Z-score normalization method. Its formula is

(1) $$\begin{array}{c} z=\frac{x-\mu }{\sigma } \end{array}$$

where *x* is the original data input amount; *z* is the normalized data output; and μ and σ represent the mean and variance of each dimension of the original dataset, respectively.

For discrete data, we use One-Hot coding for processing, which can expand the data features. It not only improves the nonlinear capability of the algorithm model, but also does not require normalization of multiple parameters.

### 3.3. The Proposed Algorithm

The basic idea of ILECA is to use the similarity measure function of high-dimensional data space as the weight to improve the between-class scatter matrix. At the same time, ILECA combines with LDA to maximize the between-class distance and minimize the within-class distance, so as to obtain the optimal transformation matrix and reduce the dimensions of the original data. Finally, ILECA combines with the ELM classification algorithm to classify the data and determine the security of the IoT devices.

Given a set *D* containing *N* train samples, $D=\left\{x_{k},t_{k}\right\},k=1,2,\cdots ,N$. Suppose $x_{ij}\in \left\{x_{k}\right\},t_{ij}\in \left\{t_{k}\right\},i=1,2,\cdots ,c,j=1,2,\cdots ,n_{i}$, $x_{ij}$ is the *j*-th sample feature vector of the *i*-th class, and $t_{ij}$ is the sample label corresponding to $x_{ij}$, where the sample feature is *d* dimension, then the total sample feature matrix can be expressed as $X^{N\times d}$; the sample has a total of *c* types; and $n_{i}$ represents the number of *i* class of samples, i.e., $N=\sum_{i=1}^{c}n_{i}$.

The total sample mean vector *u* and the class mean vector $u_{i}$ of the *i*-th sample are, respectively,

(2) $$u=\frac{1}{N}\sum_{i=1}^{c}\sum_{j=1}^{n_{i}}x_{ij}$$

(3) $$u_{i}=\frac{1}{n_{i}}\sum_{j=1}^{n_{i}}x_{ij}$$

Moreover, the within-class scatter matrix, between-class scatter matrix, and transformation matrix objective functions are defined as follows.

**Definition** **1.** *The within-class scatter matrix $S_{w}$ is expressed as*

(4) $$S_{w}=\sum_{i=1}^{c}\sum_{j=1}^{n_{j}}\left(x_{ij}-u_{i}\right){\left(x_{ij}-u_{i}\right)}^{T}$$

*The within-class scatter matrix is the mean square error of the distance between each class of sample with its center, and represents the degree of dispersion of the same class of sample.*

**Definition** **2.** *The between-class scatter matrix $S_{b}$ is expressed as*

(5) $$S_{b}=\sum_{i=1}^{c-1}\sum_{j=i+1}^{c}f_{ij}n_{i}n_{j}\left(\mu_{i}-\mu_{j}\right){\left(\mu_{i}-\mu_{j}\right)}^{T}$$

(6) $$f_{ij}=\sum_{k=1}^{d}\frac{1}{1+\left|u_{i,k}-u_{j,k}\right|}$$

*where $f_{ij}$ is a high-dimensional data spatial similarity measurement function, which represents the spatial similarity of data $\mu_{i}$ and $\mu_{j}$; $\mu_{i,k}$ and $\mu_{j,k}$ represent the mean values of data i and j in k dimensions, respectively; d is the feature dimension of data; and $n_{i}$ and $n_{j}$ represent the number of samples of class i and j, respectively.*

*The between-class scatter matrix $S_{b}$ reflects the average of the distances between the centers of various classes with different spatial similarities and the center of the total sample. $S_{b}$ represents the dispersion between classes. The range of the high-dimensional data spatial similarity measurement function is (0, 1].*

**Definition** **3.** The objective function of the optimal transformation matrix $A^{*}$ is expressed as

(7) $$\begin{array}{c} A^{*}=\arg\max_{A}\frac{A^{T}S_{b}A-A^{T}S_{w}A}{A^{T}IA}\\ \;\;=\arg\max_{A}\frac{A^{T}\left(S_{b}-S_{w}\right)A}{A^{T}IA} \end{array}$$

where A is the projection matrix and I is the identity matrix. According to the extreme value of generalized Rayleigh quotient, calculate the eigenvectors $a_{1},a_{2},\cdots ,a_{m}$ corresponding to the first m eigenvalues $\lambda_{1}>\lambda_{2}>\cdots >\lambda_{m}$ of $I^{-1}\left(S_{b}-S_{w}\right)$, and combine them into a matrix to obtain the optimal transformation matrix $A^{*}$, m=c−1. Finally, the dimensionality-reduced sample feature vector is obtained through matrix calculation:

(8) $$y_{k}=x_{k}A^{*}$$

*where $y_{k}$ is the corresponding feature vector after the dimensionality reduction of the feature vector $x_{k}$, and the dimensionality-reduced sample feature matrix is expressed as $Y^{N\times m}$.*

*After the dimensionality reduction, N samples are obtained and transformed into sample sets with new features $D^{{}^{\prime }}=\left\{y_{k},t_{k}\right\},k=1,2,\ldots ,N$, where $y_{k}={\left[y_{k1},y_{k2},\cdots y_{km}\right]}^{T}$ is the m-dimensional feature vector of the dimensionality-reduced data, $t_{k}={\left[t_{k1},t_{k2},\cdots t_{kc}\right]}^{T}$ is the sample label, and the samples have c classes.*

**Figure 3:**
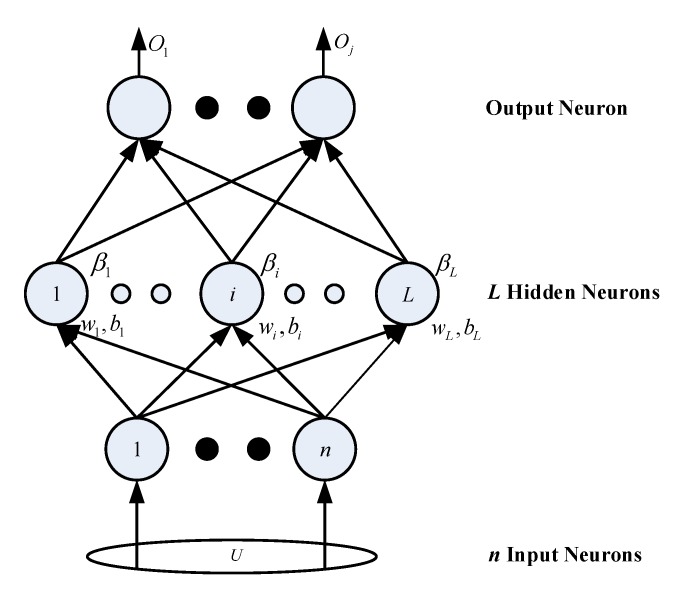
Single hidden layer neural network.

As shown in Figure 3, the new sample set obtained after dimensionality reduction is input into a single layer neural network. For the single hidden layer neural network with *L* hidden layer nodes, it can be expressed as

(9) $$\left\{\begin{array}{c} \sum_{i=1}^{L}\beta_{i}g\left(w_{i}^{T}\cdot y_{1}+b_{i}\right)=o_{1}\\ \sum_{i=1}^{L}\beta_{i}g\left(w_{i}^{T}\cdot y_{2}+b_{i}\right)=o_{2}\\ \vdots \\ \sum_{i=1}^{L}\beta_{i}g\left(w_{i}^{T}\cdot y_{N}+b_{i}\right)=o_{N} \end{array}\right.$$

where $w_{i}={\left[w_{i1},w_{i2},\cdots ,w_{im}\right]}^{T}$ is the input weight between the *i*-th hidden layer node and the input layer node, $b_{i}$ is the offset of the *i*-th hidden layer node, $\beta_{i}$ is the output weight between the *i*-th hidden layer node and the output layer node, $g\left(x\right)$ is the activation function, and $w_{i}^{T}\cdot y_{k}$ is the inner product of $w_{i}^{T}$ and $y_{k}$. The input weight $w_{i}$ and offset $b_{i}$ in the function are random numbers between (−1, 1) or (0, 1).

To minimize the output error and the label error of the corresponding sample data, an objective function is established as

(10) $$\sum_{k=1}^{N}∥o_{k}-t_{k}∥=0$$

which is

(11) $$\left\{\begin{array}{c} \sum_{i=1}^{L}\beta_{i}g\left(w_{i}^{T}\cdot y_{1}+b_{i}\right)=t_{1}\\ \sum_{i=1}^{L}\beta_{i}g\left(w_{i}^{T}\cdot y_{2}+b_{i}\right)=t_{2}\\ \vdots \\ \sum_{i=1}^{L}\beta_{i}g\left(w_{i}^{T}\cdot y_{N}+b_{i}\right)=t_{N} \end{array}\right.$$

The above *N* equations can be expressed by a matrix as

(12) Hβ=T

where

(13) $$H\left(w_{1},\cdots ,w_{L},b_{1},\cdots ,b_{L},y_{1},\cdots ,y_{N}\right)={\left[\begin{array}{ccc} g(w_{1}^{T}\cdot y_{1}+b_{1}) & \cdots & g(w_{L}^{T}\cdot y_{1}+b_{L})\\ \vdots & \ddots & \vdots \\ g(w_{1}^{T}\cdot y_{N}+b_{1}) & \cdots & g(w_{L}^{T}\cdot y_{N}+b_{L}) \end{array}\right]}_{N\times L}$$

*H* is the output matrix of the hidden layer nodes, β is the output weight matrix, and *T* is the expected output.

According to Equation (13), as long as the input weight $w_{i}$ and the offset $b_{i}$ are randomly determined, the output matrix *H* is uniquely determined. The Moore–Penrose generalized inverse matrix $H^{†}$ of *H* is used to analyze and determine the least-squares solution β of the smallest norm [25,26]

(14) $$\left\{\begin{array}{c} \beta =H^{†}T=H^{T}{\left(\frac{I}{C}+HH^{T}\right)}^{-1}T,N\leq L\\ \beta =H^{†}T={\left(\frac{I}{C}+H^{T}H\right)}^{-1}H^{T}T,L<N \end{array}\right.$$

It can be seen from the Equation (14), to obtain better generalization, the positive value IICC is added to the diagonal of $HH^{T}$ or $H^{T}H$. Then, it can repair the matrix and ensure that it is a full rank matrix. Therefore, the classifier training process is given as follows.

- **Step 1**: randomly generate the input weight $w_{i}$ and the offset $b_{i}$ of the hidden layer node i=1,2,⋯L.
- **Step 2**: calculate the hidden layer output matrix H according to Equation (13).
- **Step 3**: calculate the optimal output weight β according to Equation (14).

**Figure 4:**
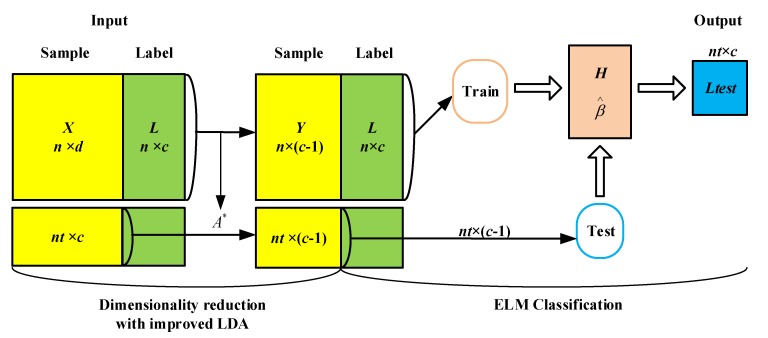
Algorithm framework.

As shown in Figure 4, we reduce the train data to generate a transformation matrix $A^{*}$, and input the dimensionality-reduced train data into the ELM classifier to calculate the final weight β. Then, let input the dimensionality-reduced test data to the ELM classifier for classification, and finally output the prediction results of the test data.

**Figure 5:**
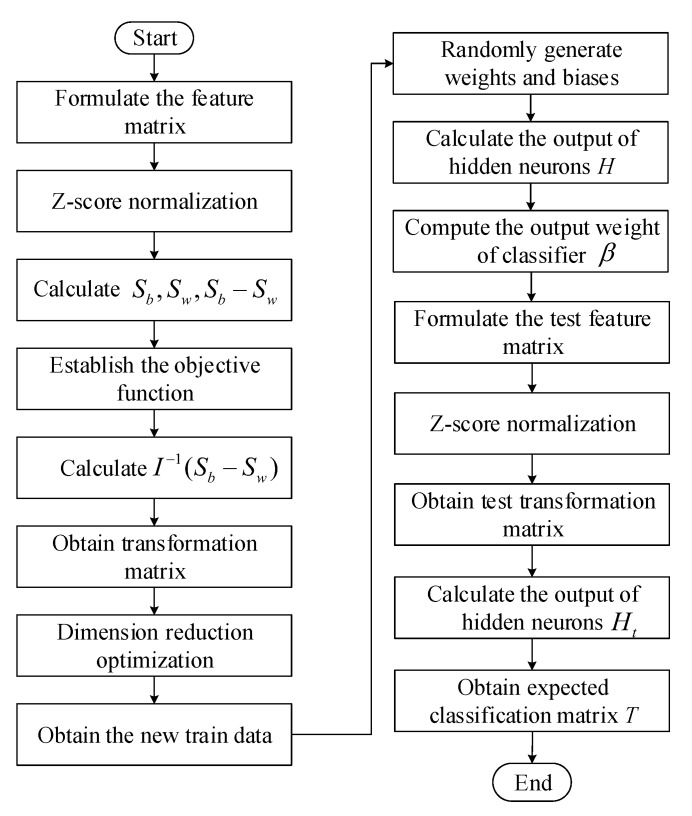
The flow chart of ILECA.

Figure 5 shows the flow chart of ILECA. The specific process of ILECA is described as follows, and the ILECA pseudocode is shown in Algorithm 1.

**Algorithm 1:** ILECA**Input:** train set $D=\left\{x_{k},t_{k}\right\},k=1,2,\ldots ,N$, test set $DT=\left\{Tx_{k},Tt_{k}\right\},k=1,2,\ldots ,n_{t}$ **Output:**
expected classification matrix *T*
 1:formulate the feature matrix *X* for *D* 2:X=Z−score(X) 3:calculate $S_{b},S_{w},S_{b}-S_{w}$ 4:obtain $A^{*}$ by solving the eigenproblem of $I^{-1}\left(S_{b}-S_{w}\right)$ 5:calculate $Y=XA^{*}$, obtain the new train data $D^{{}^{\prime }}=\left\{y_{k},t_{k}\right\}$ 6:generate $w_{i}$ and $b_{i}$ randomly, set the number of hidden neurons *L* 7:calculate the output of hidden neurons *H* according to the Equation (13) 8:calculate the output weight of classifier β according to the Equation (14) 9:formulate the feature matrix $X_{t}$ for DT10:$X_{t}=Z-score\left(X_{t}\right)$11:$Y_{t}=X_{t}A^{*}$12:calculate the output of hidden neurons $H_{t}$ for test data according to the Equation (13)13:$T=H_{t}\beta$ according to the Equation (12)14:**return***T*

- **Step 1**: Perform Z-score normalization on the train samples according to Equation (1).
- **Step 2**: Calculate the within-class scatter matrix $S_{w}$ according to Equation (4), and calculate the between-class scatter matrix $S_{w}$ according to Equation (5).
- **Step 3**: Establish the objective function according to Equation (7), calculate $I^{-1}\left(S_{b}-S_{w}\right)$, and decompose the characteristic problem to obtain the eigenvalues and eigenvectors. Take the eigenvectors corresponding to the first *m* largest eigenvalues as the transformation matrix $A^{*}$, m=c−1.
- **Step 4**: Calculate $Y=XA^{*}$ according to Equation (8), and obtain the new train data $D^{{}^{\prime }}=\left\{y_{k},t_{k}\right\}$.
- **Step 5**: Generate $w_{i}$ and $b_{i}$ randomly, and set the number of hidden neurons *L*.
- **Step 6**: Calculate the output of hidden neurons *H* according to the Equation (13).
- **Step 7**: Calculate the output weight of classifier β according to the Equation (14).
- **Step 8**: Calculate $Y_{t}=X_{t}A^{*}$.
- **Step 9**: Calculate the output of hidden neurons $H_{t}$ for test data according the to Equation (13).
- **Step 10**: Calculate the output for test data by Equation (12) with $H_{t}$ and β.

## 4. Performance Evaluation

### 4.1. Experimental Set-Up

In this paper, we use MATLAB (MathWorks, Boston, MA, USA) 2015a platform for simulation experiments.

(1) **dataset**

To verify the effectiveness of the intrusion detection algorithm in IoT application, we choose NSL-KDD [27] as the dataset in our experiments. As there is currently a lack of public datasets for IoT intrusion detection, NSL-KDD can still serve as an effective benchmark dataset to evaluate different intrusion detection methods and related security studies.

NSL-KDD is actually an intrusion detection project by the defense advanced research projects agency (DARPA) at MIT’s Lincoln laboratory. It mainly simulates the network environment of air force LAN, which collects TCP network connection data and system audit data through experiments, as well as simulates various users, network traffic, and attack types. Specifically, it is divided into attack data and normal network traffic data. Among them, the attack data are denial-of-service (DOS), unauthorized access from a remote machine (R2L), unauthorized access to local superuser (root) privileges (U2R), and surveillance and other probing (PROBING), including a total of 39 attack types. The features in NSL-KDD to simulate IDS are shown in Table 2 in detail.

We select the training set containing 22 subdivided attack types and the test set containing 39 subdivided attack types for the experiment. The reason we use these two datasets with different numbers of attack types is to evaluate the generalization ability of the proposed algorithm. Because the ability to detect unknown attack types is also an important indicator of the pros and cons of intrusion detection algorithms, the experiment randomly selects 20,000 data samples as the experimental train set and 2000 data samples as the experimental test sample. To avoid randomness of the results and prove the generalization, we conduct 50 random sampling experiments, and finally obtain the average of the evaluation results.

(2) **Evaluation criteria and comparison algorithm**

To verify the performance of ILECA, we evaluate the algorithm from the time complexity and prediction accuracy, where the time complexity is represented by the algorithm runtime. As an important evaluation criterion for algorithm performance, the accuracy of the prediction is represented by accuracy, detection rate, and false detection rate (including false detection rate of normal class and false detection rate of attack class).

- **Accuracy**: The proportion of samples predicted to be correct.
- **Detection rate**: The proportion of the number of attack samples that are correctly detected to the total number of attack samples.
- **False detection rate of normal class**: The proportion of the normal class samples that are falsely detected as attack classes to the total number of normal class samples.
- **False detection rate of attack class**: The proportion of the attack class samples that are falsely detected as normal classes to the total number of attack class samples.

To prove the superiority of our proposed algorithm, we compare our algorithm with the ELM algorithm [21], VNELM algorithm [28], PCA-ELM algorithm [29], LDA-ELM algorithm, and EGRNN algorithm [20]. The experiment is also performed in terms of efficiency, accuracy, detection rate, and false detection rate.

### 4.2. Meta-Parameter Analysis

According to the parameter distribution of the extreme learning machine [30], the main influencing factors of ILECA are the constant parameter *C*, the number of hidden layer nodes *L*, and the activation function. Among them, we take the number of hidden layer nodes as a variable for experiment, so as to select the optimal parameter *L*. The selection of the activation function mainly depends on the empirical judgment of the input data. We use the number of hidden layer nodes *L* as a variable to compare the performance of the algorithms under different activation functions and different parameters *C*. Then, we select the better meta-parameters for subsequent comparison experiments. We set the number of hidden layer nodes as L∈{20,40,⋯,200}, parameter $C\in \{2^{-20},2^{-15},\cdots ,2^{20}\}$, and activation function $g\left(x\right)\in {\{}^{\prime }sigmoid^{\prime }{,}^{\prime }sin^{\prime }{,}^{\prime }rbf^{\prime }{,}^{\prime }hardlim^{\prime }\}$. Among the four activation functions, the sigmoid function is an exponential function that maps data to (0, 1), the sin function is a sine function, rbf is a radial basis function, and hardlim is a threshold transfer function.

**Figure 6:**
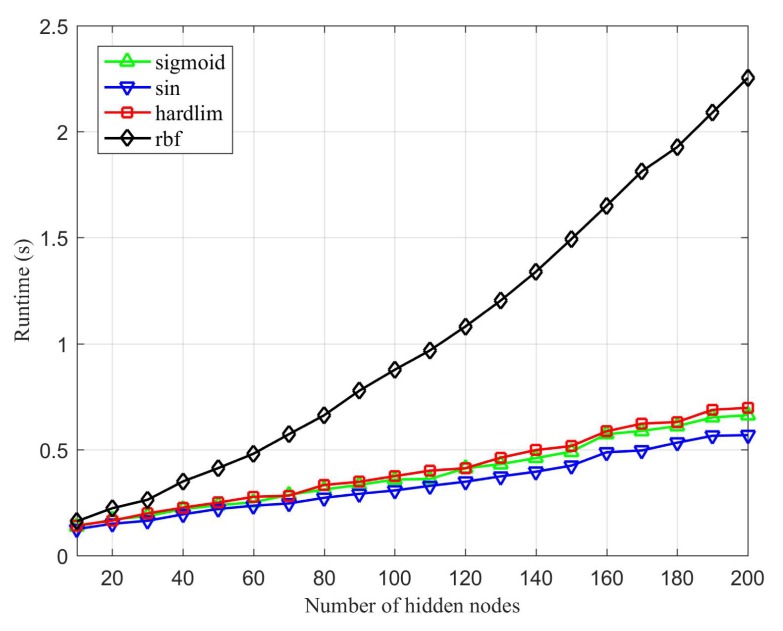
Runtime of ILECA under different activation functions.

Figure 6 shows the runtime of ILECA under four different activation functions and different numbers of hidden layer nodes. We can see that the running efficiency of the proposed ILECA under the three activation functions of sigmoid, sin, and hardlim is similar, whereas it requires more runtime under the rbf activation function.

**Figure 7:**
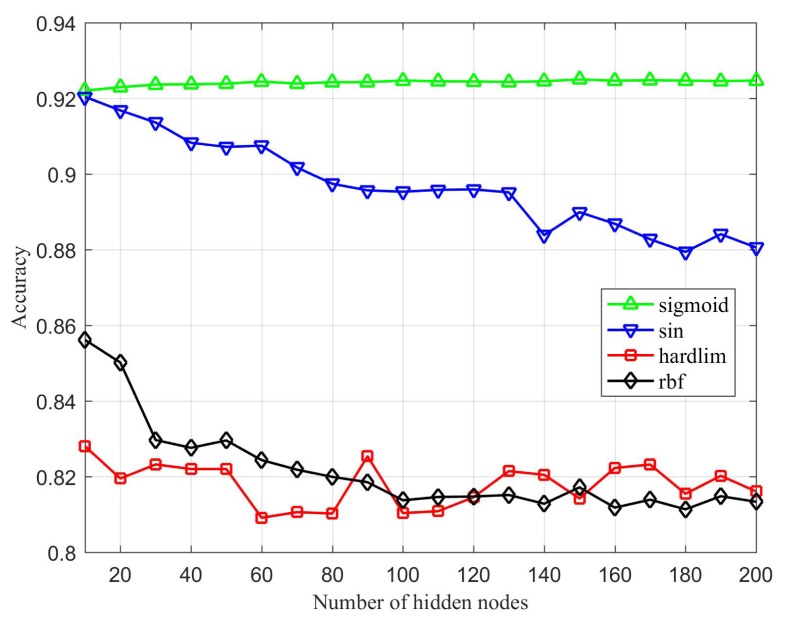
Accuracy of ILECA under different activation functions.

**Figure 8:**
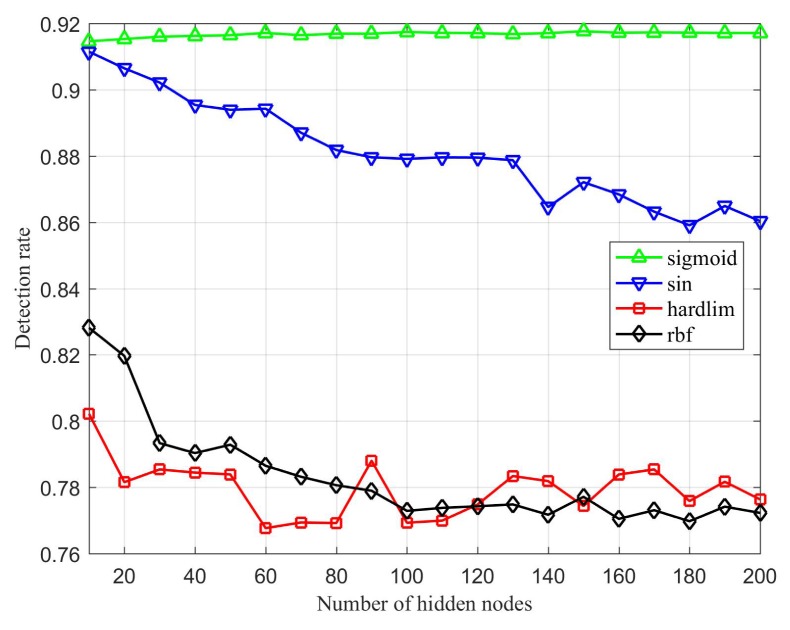
Detection rate of ILECA under different activation function.

**Figure 9:**
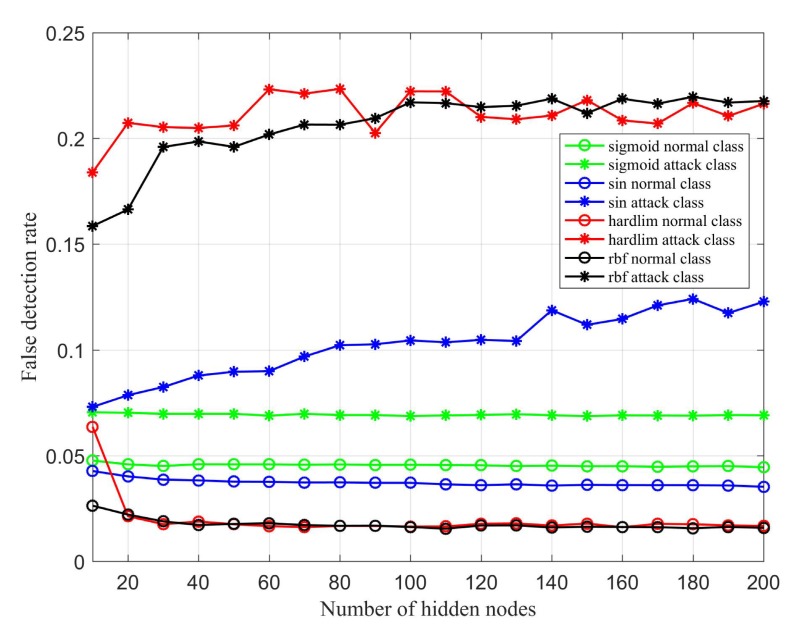
False detection rate of ILECA under different activation functions.

Figure 7, Figure 8 and Figure 9 represent the comparison of the algorithm’s prediction performance under different activation functions with different numbers of hidden layer nodes. First, from the comparison of the accuracy rates in Figure 7, it can be seen that ILECA has the best performance under the sigmoid function. Under the sigmoid function, as the number of hidden layer nodes increases, the test results show a slight upward trend, and the increase is gentle. Under the sin function, the effect is best when the number of hidden layer nodes is 10, but it is still inferior to sigmoid. After that, the accuracy of the algorithm decreases as the number of hidden layer nodes increases. When the activation function is the hardlim function or rbf function, the accuracy of the algorithm is low. Figure 8 is an evaluation of the attack detection rate. We can find that the performance of ILECA under different activation functions is similar to the accuracy rate. When the activation function is sigmoid function, ILECA has the best performance, followed by the sin function. The hardlim function and rbf function perform relatively poorly, and the results fluctuate greatly under different numbers of hidden layer nodes.

The false detection rate shown in Figure 9 includes the false detection rate of the normal class and the false detection rate of the attack class. When the number of hidden layer nodes is 20, according to the false detection rate of the normal class, the rbf function has the best performance, followed by the hardlim function. The performances of the sin function and the sigmoid function are relatively close, and the false detection situation is slightly worse among the four. According to the false detection rate of attack class, the sigmoid function is the best, and the sin function is the second, but rbf function has the highest false detection rate. When the number of hidden layer nodes is greater than 20 and continuously increases, the hardlim function’s false detection rate of normal class becomes lower, and the performance of the rbf function is similar and relatively stable. The performance of sin function and sigmoid function continue to decrease, but they are still higher than the other two. According to the false detection rate of the attack class, with the increase of the number of hidden layer nodes, the false detection rate of the sin function and sigmoid function generally increases, and the algorithm performance decreases, whereas the hardlim and rbf functions fluctuate greatly, and the rbf function’s false detection rate of the attack class is highest.

Based on the above analysis, the performance of the sigmoid activation function in terms of evaluation indicators other than the false detection rate of the normal class is optimal. Consequently, we determine the sigmoid function as the optimal activation function of the ILECA.

Under the sigmoid function, as the number of hidden layer nodes increases, each accuracy index changes, and the runtime increases accordingly. Therefore, to select the optimal number of hidden layer nodes *L*, we use the ranking method, namely, technique for order of preference by similarity to ideal solution(TOPSIS) [31].

Table 3 shows the TOPSIS proximity of the number of nodes in the different hidden layers under the sigmoid activation function. According to these proximity values, we can conclude that the proximity is highest when the number of hidden layer nodes L=20, so we choose 20 hidden layer nodes as the proposed intrusion detection algorithm parameter for comparative experiments.

Table 4 shows the TOPSIS proximity under different parameters *C*. According to these proximities, the proximity is very similar when *C* is $2^{-10}$, $2^{-5}$, and $2^{-0}$, and the proximity is highest when *C* is $2^{-5}$. Thus, we choose $C=2^{-5}$ as the final parameter.

In summary, we choose the sigmoid function as the activation function, L=20 as the number of hidden layer nodes, and $2^{-5}$ as the parameter *C*.

### 4.3. Results and Discussion

To verify the intrusion detection performance of ILECA in the IoT environment, we compare the indicators such as runtime, accuracy, detection rate, and false detection rate with other algorithms to prove the superiority of ILECA in time efficiency and detection accuracy.

**Figure 10:**
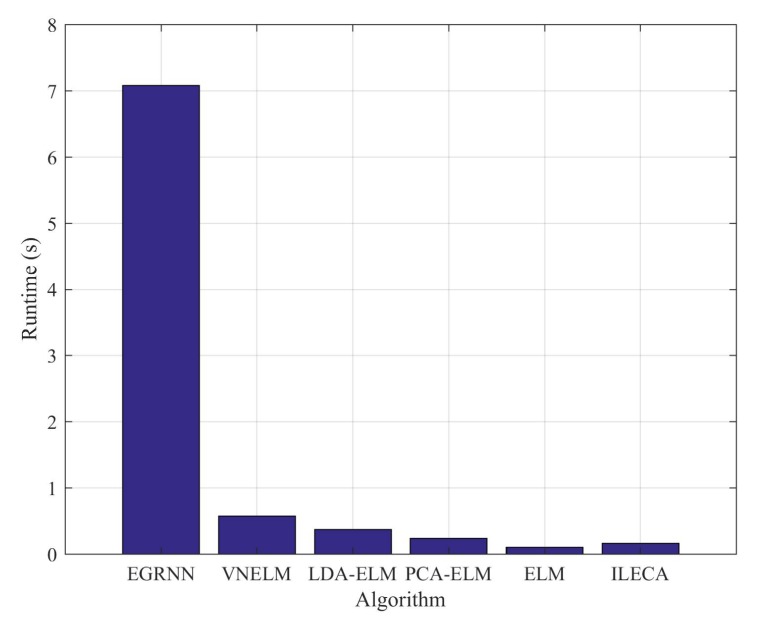
Runtime of algorithms.

As shown in Figure 10, the time efficiency of the algorithms using extreme learning machines as intrusion detection classifiers is very high. Compared with the EGRNN algorithm, the algorithm based on the ELM classifier greatly improves the calculation efficiency through the weights and offset of the random neural network. The runtime of our proposed ILECA is only lower than that of the ELM algorithm. Considering the ELM algorithm directly classifies the data without preprocessing, its running efficiency is the highest. Compared with other algorithms, our ILECA simplifies and improves the solution of the transformation matrix in LDA and the solution of output weight β in ELM, which reduces the computational time complexity and improves the operation efficiency.

**Figure 11:**
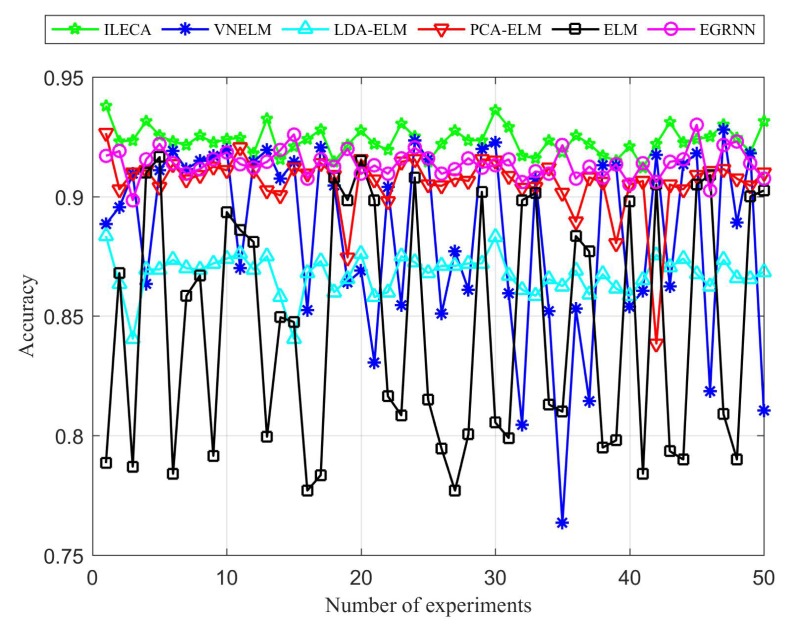
Accuracy of algorithms.

**Figure 12:**
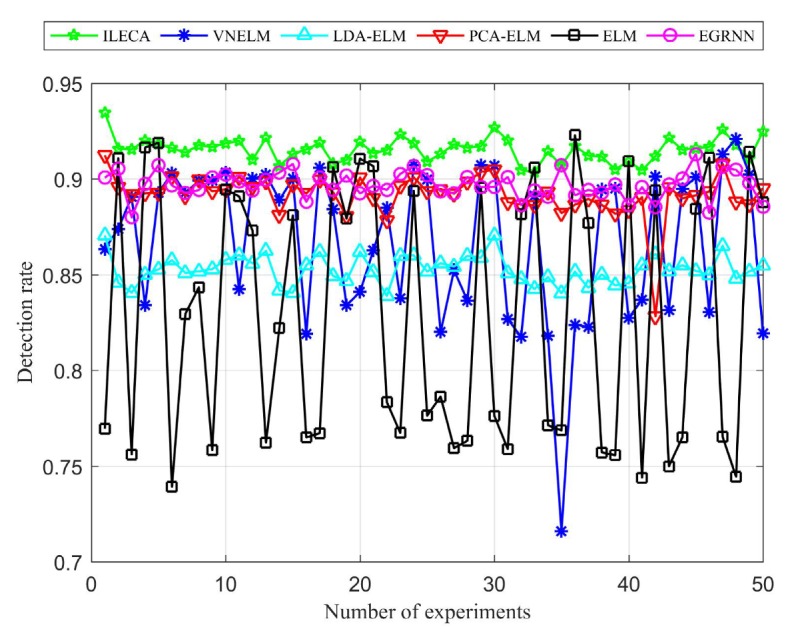
Detection rate of algorithms.

Figure 11 and Figure 12 are comparisons of the accuracy and detection rates of the algorithms. We can see that the ILECA, EGRNN, PCA-ELM, and LDA-ELM algorithms all have good stability, whereas ELM and VNELM algorithms have less stability, especially ELM algorithm. This is because each experimental data is randomly selected and the ELM algorithm does not perform any preprocessing of the data, which makes the results of each time fluctuate greatly. The VNELM algorithm incorporates a variety of preprocessing methods. There is a certain difference in the adaptability of the data form between different methods, which also leads to unstable results. In addition, compared with other algorithms, the ILECA performs almost optimally in the performance of multiple experiments. As a result, it indicates that the ILECA has a good generalization ability.

**Figure 13:**
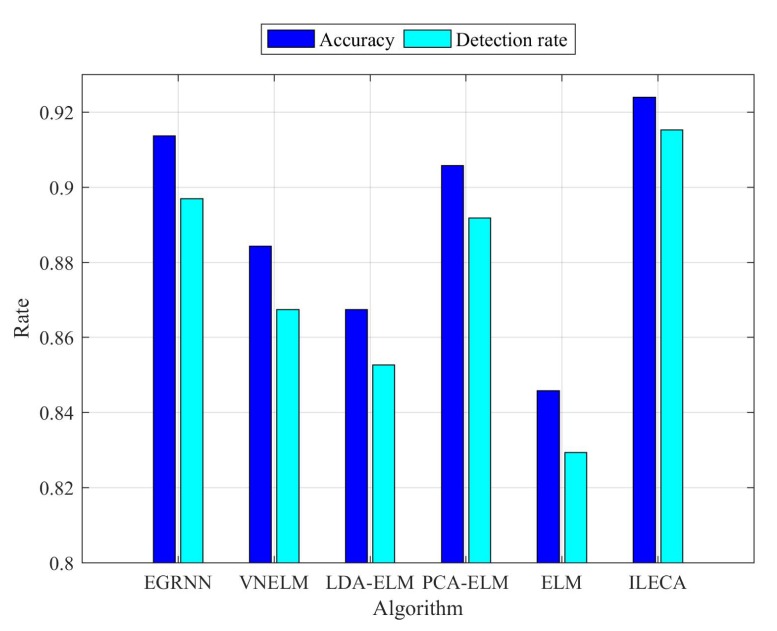
Accuracy and detection rate.

As shown in Figure 13 and Table 5, the accuracy of ILECA is 92.35%, which is higher than the other five comparison algorithms. Similarly, the detection rate of ILECA is up to 91.53%, which is the highest value within all algorithms. Among them, the detection rate and accuracy of ILECA are slightly higher than the EGRNN algorithm. This indicates that compared with the traditional neural network algorithm, ILECA can guarantee the accuracy of detection while ensuring higher time efficiency. Compared with the other algorithms, the ELM algorithm without any processing performs the worst among all algorithms, and its accuracy is 84.59% and its detection rate is only 82.94%. Meanwhile, ILECA still has better performance than the VNELM, PCA-ELM, and LDA-ELM algorithms, which also perform dimensionality reduction feature extraction. This is because ILECA expands the distance between similar classes through data projection to obtain new data dimensions, and then classifies the dimensionality-reduced data to improve the accuracy and detection rate of the samples.

**Figure 14:**
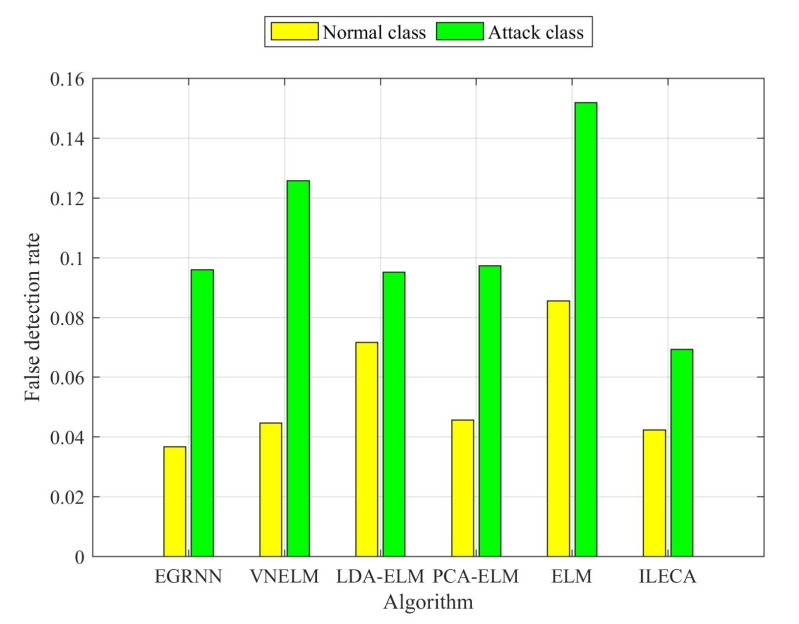
False detection rates.

Subsequently, as shown in Figure 14 and Table 5, according to the false detection rate of the normal class, ILECA has similar performance to the VNELM and PCA-ELM algorithms, and its false detection rate of the normal class is 4.24%. Moreover, ILECA is significantly better than the LDA-ELM and ELM algorithms, which are slightly inferior to the EGRNN algorithm. The EGRNN algorithm has the best false detection rate of the normal class, that is, 3.67%, but its runtime is much higher than other algorithms. On the other hand, according to the false detection rate of the attack class, ILECA performs better than other algorithms; its false detection rate for the attack class is 6.93%. Obviously, ILECA has a higher ability to identify attack classes. For IoT security, the harmfulness caused by an intrusion detection algorithm that determines an attack class to be a normal class is greater than the normal class as an attack class, so ILECA is more suitable for IDS. In addition, the runtime of ILECA is 0.1632s, which is relatively better than most of the compared algorithms. ELM algorithm has the best runtime, i.e., 0.1036s, but its other performance is the worst within all the algorithms.

Based on the above analysis and comparison of various algorithms, we have found that ILECA has better time efficiency, detection performance, and generalization. Therefore, ILECA is a better choice for the IoT intrusion detection than other algorithms.

## 5. Conclusion and Future Work

Aiming at the problem of intrusion detection in IoT, we propose an improved LDA-based ELM classification for the intrusion detection algorithm ILECA. The ILECA first uses the improved linear discriminant analysis to reduce the feature dimensions of the data, and then uses the extreme learning machine algorithm as the classifier. Finally, we verify the performances and efficiency of ILECA through experiments on the NSL-KDD dataset while comparing it with the other five algorithms. The results show our ILECA has the best accuracy and detection rate, which are 92.35% and 91.53%, respectively, but the runtime is only 0.1632 s. Therefore, ILECA has good generalization and real-time characteristics, of which its overall performance is better than the other five typical algorithms.

Although the proposed IDS has verified its effectiveness through simulation experiments, it has not been put into practical IoT. Therefore, combining algorithms and hardware to practical applications is the aim of our future work. In addition, the reliability of IoT devices is also an important indicator in determining the security of IoT devices. Consequently, in the future, we can further use the reputation evaluation to improve the security of the IoT. 

## Figures and Tables

**Table 1:** The correlated variables of the intrusion detection algorithm (ILECA).

Variable	Description
*D*	training set
$x_{ij}$	the *j*-th sample feature vector of the *i*-th class
$t_{ij}$	sample label corresponding to $x_{ij}$
$S_{w}$	within-class scatter matrix
$S_{b}$	between-class scatter matrix
$f_{ij}$	high-dimensional data spatial similarity measurement function
$A^{*}$	optimal transformation matrix
$D^{{}^{\prime }}$	dimensionality-reduced training set
$w_{i}$	input weight between the *i*-th hidden layer node and the input layer node
$b_{i}$	offset of the *i*-th hidden layer node
*H*	output matrix of the hidden layer nodes
β	output weight matrix
*T*	expected output

**Table 2:** The features of NSL-KDD used to simulate the intrusion detection system (IDS).

Type	Name	Description	Numerical Type
Basic	duration	connection duration	continuous
	protocol_type	protocol type	discrete
	service	targeted network service type	discrete
	src_bytes	number of bytes sent from source to destination	continuous
	dst_bytes	number of bytes sent from destination to source	continuous
	flag	the connection is normal or not	discrete
	land	whether the connection is from/to the same host/port	discrete
	wrong_fragment	number of “wrong” fragment	continuous
	urgent	number of urgent packets	continuous
Traffic	count	number of connections to the same host in the first two seconds	continuous
	serror_rate	“SYN” error on the same host connection	continuous
	rerror_rate	“REJ” error on the same host connection	continuous
	same_srv_rate	number of of same service connected to the same host	continuous
	diff_srv_rate	number of of different services connected to the same host	continuous
	srv_count	number of connections to the same service in the first two seconds	continuous
	srv_serror_rate	“SYN” error on the same service connection	continuous
	srv_rerror_rate	“REJ” error on the same service connection	continuous
	srv_diff_host_rate	number of different targeted host connected to the same service	continuous

**Table 3:** Technique for Order of Preference by Similarity to Ideal Solution (TOPSIS) proximity under different hidden layer node numbers.

*L*	TOPSIS Proximity	*L*	TOPSIS Proximity
10	0.8933	110	0.5744
**20**	**0.9154**	120	0.4798
30	0.9019	130	0.4450
40	0.8368	140	0.3913
50	0.8033	150	0.3379
60	0.7788	160	0.1923
70	0.7140	170	0.1727
80	0.6663	180	0.1361
90	0.6261	190	0.0924
100	0.5795	200	0.1041

**Table 4:** TOPSIS proximity under different *C*.

*C*	TOPSIS Proximity	*C*	TOPSIS Proximity
$2^{-20}$	0.4272	$2^{5}$	0.6406
$2^{-15}$	0.4247	$2^{10}$	0.6171
$2^{-10}$	0.7210	$2^{15}$	0.5681
$2^{-5}$	**0.7246**	$2^{20}$	0.6046
$2^{-0}$	0.7239		

**Table 5:** Algorithm performance.

Algorithm	Accuracy	Detection Rate	False Detection Rate of Normal Class	False Detection Rate of Attack Class	Runtime(/s)
VNELM	88.43%	86.74%	4.47%	12.58%	0.5788
LDA-ELM	86.74%	85.27%	7.16%	9.51%	0.3732
PCA-ELM	90.58%	89.18%	4.56%	9.73%	0.2381
ELM	84.59%	82.94%	8.55%	15.19%	**0.1036**
EGRNN	91.37%	89.70%	**3.67%**	9.60%	7.0790
ILECA	**92.35%**	**91.53%**	4.24%	**6.93%**	0.1632
